# Statistical optimization of hyaluronic acid enriched ultradeformable elastosomes for ocular delivery of voriconazole via Box-Behnken design: *in vitro* characterization and *in vivo* evaluation

**DOI:** 10.1080/10717544.2020.1858997

**Published:** 2020-12-21

**Authors:** Abdurrahman Muhammad Fahmy, Mariam Hassan, Doaa Ahmed El-Setouhy, Saadia Ahmed Tayel, Abdulaziz Mohsen Al-Mahallawi

**Affiliations:** a Department of Pharmaceutics and Industrial Pharmacy, Faculty of Pharmacy, Cairo University, Cairo, Egypt; b Department of Microbiology and Immunology, Faculty of Pharmacy, Cairo University, Cairo, Egypt; c Department of Pharmaceutics, Faculty of Pharmacy, October University for Modern Sciences and Arts (MSA), Giza, Egypt

**Keywords:** Voriconazole, modified ethanol injection method, hyaluronic acid, Brij S100 ocular safety, antifungal susceptibility testing

## Abstract

Voriconazole (VCZ) is a well-known broad spectrum triazole antifungal, mainly used orally and intravenously. The study aimed to formulate VCZ into ultradeformable elastosomes for the topical treatment of ocular fungal keratitis. Different formulae were prepared using a modified ethanol injection method, employing a 3^3^ Box-Behnken design. They were characterized by measuring their entrapment efficiency (EE%), particle size (PS), polydispersity index (PDI) and zeta potential (ZP). The optimized formula was subjected to further *in vitro* investigations and *in vivo* evaluation studies. The prepared vesicles had satisfactory EE%, PS, PDI and ZP values. The numerical optimization process suggested an optimal elastosomal formula (OE) composed of phosphatidyl choline and brij S100 at the weight ratio of 3.62: 1, 0.25%w/v hyaluronic acid and 5% (percentage from phosphatidyl choline/brij mixture) polyvinyl alcohol. It had high EE (72.6%), acceptable PS and PDI (362.4 nm and 0.25, respectively) and highly negative ZP of −41.7 mV. OE exhibited higher elasticity than conventional liposomes, with acceptable stability for three months. Transmission electron microscopy demonstrated the spherical morphology of vesicles with an external transparent coat of Hyaluronic acid. OE was expected to cause no ocular irritation or blurring in vision as reflected by pH and refractive index measurements. The histopathological study revealed the safety of OE for ocular use. The fungal susceptibility testing using *Candida albicans* demonstrated the superiority of OE to VCZ suspension, with greater and more durable growth inhibition. Therefore, OE can be regarded as a promising formula, achieving both safety and efficacy.

## Introduction

Treatment of ocular fungal infections is one of the most difficult challenges in the field of ophthalmology (Deibel & Cowling, 2013). Most cases appear in hot and humid areas characterized by low socioeconomic levels and poor hygiene (Thomas & Kaliamurthy, 2013). In addition, the eye is a small complex organ with small volume of tear fluid available for ocular drug retention and absorption. The tear film turnover and blinking of the eyes reduce the effective fraction of the applied dose available for absorption. The remaining fraction of the dose then must permeate through the tight junctions of corneal epithelium to be absorbed and provide therapy (Gaudana et al., 2010; de Sá et al., 2015; Liu et al., 2016; Alharbi et al., 2020). Moreover, fungal infections of the eye show delayed onset of symptoms, then they become more difficult to treat (Khater et al., 2014). Treatment options in these cases are usually invasive e.g. the use of penetrating keratoplasty (Mandell & Colby, 2009) or necessitate the use of high dose of systemic and topical antifungals, finally leading to noncompliance of the patients (Tilak et al., 2010; Vanzzini et al., 2010). Here appears the need to use simpler and more effective approaches to manage the situation.

Voriconazole (VCZ), developed by modifying the molecule of fluconazole, is the first available second-generation triazole antifungal. It has potent and broad spectrum efficacy against numerous fungal species, including *Aspergillus, Candida* and other fungi with low minimal inhibitory concentrations (Marangon et al., 2004; Theuretzbacher et al., 2006). VCZ is commercially available in oral and intravenous dosage forms. However, the systemic administration of VCZ is associated with serious adverse effects and drug interactions (Theuretzbacher et al., 2006). VCZ has molecular weight of 349.3 and *log P* value of 1.8 making it a suitable candidate for ocular drug delivery with good corneal permeation (de Sá et al., 2015; Veloso et al., 2018). However, no commercial ocular product of VCZ is available yet, possibly due to the limited aqueous solubility of VCZ. The reconstituted lyophilized powder of cyclodextrin-VCZ complex for intravenous injection is instilled into the eye of patients for topical therapy of fungal keratitis. This should be made so frequently (every 1–2 h) to achieve therapeutic efficacy, but finally leads to the patients being non-compliant (de Sá et al., 2015).

Recently, some efforts have been made to formulate VCZ using nanotechnology. Kumar and Sinha formulated VCZ in microemulsions that had a small droplet size (<200 nm) with sustained release pattern and enhanced permeation over 12 h (Kumar & Sinha, 2014). The same researchers formulated VCZ in solid lipid nanoparticles utilizing compritol, soy lecithin, poloxamer 188 and sodium taurocholate, to achieve high entrapment efficiency (EE %) of 64.13% and small particle size (PS) of 139.1 nm (Kumar & Sinha, 2016). Okur et al. (2019) incorporated VCZ into thermo-sensitive *in situ* gels using poloxamers 407 and 188, along with carboxymethyl cellulose. The formulations had satisfactory gelation temperatures, pH values, clarity and spreadability. de Sá et al. (2015) utilized both conventional and cationic liposomes to entrap VCZ. The formulae contained soy phosphatidylcholine containing or not 1,2-dioleoyl3-trimethylammonium-propane (DOTAP) and cholesterol and were characterized by high EE %, small PS and low levels of irritation. Shukr entrapped VCZ in niosomes composed of span 60 and pluronic L64, followed by incorporation in alginate in situ gelling inserts that increased the drug retention in the eye and hence its aqueous humor concentration (Shukr, 2016). Andrade et al. (2016) used glyceryl behenate/capric caprylic triglyceride, polysorbate 80, sorbitan trioleate, and cetylpyridinium chloride to fabricate nanostructured lipid carriers for entrapment of VCZ. The fabricated systems had good characteristics enabling them to deliver therapeutic amounts of VCZ after 30 minutes.

The aim of the current work was to develop VCZ ultradeformable elastosomes. They are vesicular structures that depend primarily on the use of phospholipids, coupled with edge activators (EA), with or without other additives e.g. polyvinyl alcohol and hyaluronic acid, to enhance their stability and bioadhesion, respectively. This is particularly suitable for the dual nature of the corneal tissue (lipophilic epithelium + hydrophilic stroma) (Hathout et al., 2007; Hathout & Omran, 2016). These vesicles contained in aqueous vehicles, combine the advantages of good physical and chemical stability, simplicity of application and prolonged corneal contact time, to achieve safe, sustained ocular VCZ delivery and hence, therapeutic efficacy.

In general, the target of the optimization of pharmaceutical formulations is to determine the levels of variables required to produce a high-quality product. The numerical optimization process, based on the desirability criterion is usually performed using computer software, e.g. Design Expert® by choosing the limits of factors to be included and setting certain constraints for the measured responses, according to the goals of the study. The formula with highest desirability is usually chosen and prepared. The observed values of different responses should be near the predicted ones. This indicates the validity of the design (Basalious et al., 2010; Al-Mahallawi et al., 2014).

## Materials and methods

### Materials

Voriconazole (VCZ) was kindly supplied by Eva pharma, Egypt. Hyaluronic acid (HA) (supplied as sodium hyaluronate, molecular weight 400–800 kdaltons) was purchased from Acros Organics, Belgium. l-α Phosphatidyl choline from egg yolk (≈60% by TLC) (PC), polyvinyl alcohol (PVA) molecular weight 146,000–186,000 and polyoxyethylene (100) stearyl ether (brij S100) were purchased from Sigma Chemical Company, USA. Chloroform, disodium hydrogen phosphate, calcium chloride dihydrate, sodium chloride and absolute ethanol were purchased from El-Nasr Pharmaceutical Chemicals Company, Cairo, Egypt.

### Methods

#### Experimental design

VCZ-loaded ultradeformable elastosomes were prepared according to a 3^3^ Box-Behnken design using Design-Expert® software (Stat-Ease, Inc., Minneapolis, MN) to study the effect of different variables on vesicle properties. It was chosen as it aids in reducing the number of experimental runs needed for conducting the study, hence optimizing costs and saving time (Alruwaili et al., 2020; Radwan et al., 2020). Factors, their levels and desirability constraints are listed in Table 1.

**Table 1.** Box-Behnken design used for optimization of VCZ-loaded ultradeformable elastosomal formulae.

Factors (independent variables)	Levels		
	(−1)	(0)	(+1)
X_1_: PC: EA weight ratio	1:1	2.5:1	4:1
X_2_: Percentage of hyaluronic acid (% w/v)	0.25	0.5	0.75
X_3_: Percentage of polyvinyl alcohol (PVA) (%)	1	3	5

Responses (dependent variables)	Desirability constraints
Y_1_: Entrapment efficiency (EE) (%)	Maximize
Y_2_: Particle size (PS) (nm)	Minimize
Y_3_: Zeta potential (ZP) (mV)	Maximize in terms of absolute value
Y_4_: Polydispersity index (PDI)	Minimize

PC: phosphatidyl choline; EA: edge activator.

#### Preparation of VCZ ultradeformable elastosomes using modified ethanol injection method

VCZ ultradeformable elastosomes were prepared by a modified ethanol injection method (Kakkar & Kaur, 2011; Abdelbary et al., 2019). VCZ and PC were dissolved in 4 ml of a 1:1 ethanol/chloroform mixture. The organic mixture was introduced drop by drop into a hot (60° C) aqueous phase consisting of simulated tear fluid (STF) of pH 7.4 – containing 0.2 g NaHCO_3_, 0.008 g CaCl_2_.2H_2_O and 0.67 g NaCl per 100 ml – (Shastri et al., 2010) in which brij S100 (EA) and PVA were already dispersed. The volume ratio of aqueous phase to organic phase was 2.5. The formed mixture was continuously stirred on a hot plate magnetic stirrer (Model MSH-20D, Witeg Labortechnik GmbH Germany) for 30 minutes at 800 rpm to fully evaporate the organic solvents and form VCZ-loaded aqueous elastosomal dispersions. HA was then dispersed by sprinkling while stirring at 800 rpm at room temperature until a fully homogenous dispersion was obtained. The formulae were kept refrigerated (4–8 °C) in 30 ml glass vials till investigated.

#### Determination of drug content and percentage entrapment efficiency (EE %)

Ethanol was used to lyse vesicles. For the determination of drug content, 0.1 ml of the formula was added to a volumetric flask (25 ml). The volume was completed to 25 ml by ethanol, followed by sonication for 30 seconds to ensure the disruption of all vesicles. The UV absorbance was measured using spectrophotometer (model UV-1601 PC, Shimadzu, Kyoto, Japan), at the predetermined λ_max_ (256 nm). To determine EE %, 1 ml of the formula was subjected to ultracentrifugation at 22,000 rpm and 4 °C for 1 hour using cooling ultracentrifuge (model 3-30KS, Sigma Zentrifugen, Germany), followed by the separation of supernatant, suitable dilution and measurement in the same manner. The EE % was then calculated according to a previously established calibration curve from the following equation (Al-Mahallawi et al., 2017):

(1) $$\text{EE}\;\%=\left(X_{1}–X_{2}\right)\text{ x }100/X_{1}$$

Where X_1_ = Initial amount of drug and X_2_ = Amount of free drug in the supernatant.

#### Determination of particle size (PS), polydispersity index (PDI) and zeta potential (ZP)

Zetasizer (Nano ZS, Malvern Panalytical Ltd, Malvern, UK) was used to measure the PS (Z-average) and PDI at 25 °C. The formulae were adequately diluted before every measurement for obtaining the optimal intensity of light scattering (Scognamiglio et al., 2013; Abd-Elsalam et al., 2018). ZP was determined using the same equipment by tracking the mobile particles in electric field (Abd-Elsalam et al., 2018). The recorded results were the averages of triplicate experiments ± standard deviation.

#### Further *in* vitro characterization of optimal ultradeformable elastosomal formula

##### Measurement of the deformability index (DI) of optimal ultradeformable elastosomes

To demonstrate the elasticity of the prepared optimal ultradeformable elastosomes, its deformability index was measured and compared to that of a similar liposomes formula in which the EA was replaced with cholesterol (Al-mahallawi et al., 2019). Briefly, vesicles suspension was extruded under a pressure of 2.5 bar using air compressor (Haug Kompressoren AG; Büchi Labortechnik AG, Flawil, Switzerland), through cellulose acetate filters of 200 nm pore size (El Zaafarany et al., 2010; Lei et al., 2013). Deformability index (DI) was calculated from the following formula (Gupta et al., 2005):

(2) $$\mathrm{DI}=J\;{\left(\frac{r_{v}}{r_{p}}\right)}^{2}$$

where *J* is the weight of dispersion extruded in 10 minutes, *r_v_* is the size of vesicles after extrusion (nm), and *r_p_* is the pore size of the barrier (nm).

##### Effect of short-term storage

Optimal ultradeformable elastosomal formula (OE) was stored for 3 months in refrigerator 4–8 °C in sealed glass vials (30 ml) (Al-Mahallawi et al., 2014). The formula was observed for changes in visual appearance. Drug content, EE %, PS, ZP, PDI and DI were compared using Student *t* test (α was set at 0.05) before and after storage to see whether storage affected these parameters.

To demonstrate the role of PVA as a stabilizer, the optimal formula – lacking PVA – was prepared. The PS, ZP and PDI of such formula were measured when fresh and after storage for 3 months, and the mean values were compared using Student *t* test (α was set at 0.05).

##### Morphology of VCZ optimal ultradeformable elastosomes

Transmission electron microscope (TEM) was utilized for the examination of the morphology of OE. The formula was properly diluted followed by the deposition of a single drop on a carbon-coated copper grid and negative staining by 2% w/v phosphotungestic acid in water. The examination was carried out at 80 kV (Fahmy et al., 2018; Shahab et al., 2020). For comparative purposes, the optimal formula lacking HA (OE_zero_) was also visualized under the same conditions.

##### pH measurement

One aspect of testing the ocular safety of OE and OE_zero_ was to measure their pH (Fouda et al., 2018). The recorded results were the averages of triplicate experiments ± standard deviation.

##### Refractive index (RI)

The light refractive index (RI) was measured at ambient temperature using an Easy R40® automatic refractometer, supplied by Mettler Toledo (Columbus, Ohio, United States), to ensure there is minimal or no blurring in vision caused by the formula (Fouda et al., 2018).

##### *In vivo* evaluation of OE and OE_zero_ in New Zealand white rabbits

###### Animals

A total of eleven Adult male New Zealand rabbits weighing 2–3 kg, were included. Each rabbit was kept in a separate cage under standard conditions of humidity, temperature and light/dark cycling. Standard dry food and water were supplied *ad libitum*. A slit lamp was used to examine the rabbits’ eyes for any blemishes or diseases. All experiments including animals were approved by the research ethics committee of the Faculty of Pharmacy, Cairo University (PI 2689) and following the principles of the Declaration of Helsinki.

###### Histopathological evaluation

The safety of selected formulae was evaluated by comparison to a negative control (sterile normal saline) (Vargas et al., 2003). One drop from each liquid was instilled into one eye of a male New Zealand rabbit, while the other eye was reserved for the negative control. The treatments were applied twice per day for seven days (Abdelbary et al., 2016). Ketamine (200 mg/kg) and xylazine (20 mg/kg) were used for general anesthesia of rabbits. After sacrificing the animals by decapitation, corneas were excised from the separated eyes and carefully rinsed using normal saline to prevent their damage followed by storage in 10% v/v formalin saline solution till processed (Kakkar & Kaur, 2011; Dai et al., 2013; Abdelbary et al., 2016). Solid paraffin cubes enclosing the corneas were obtained by immersing the corneas in molten paraffin, followed by cooling. Thin slices were obtained using a microtome. Eosin and hematoxylin were used as staining agents. A digital light microscope (Leica, Cambridge, UK) was used for the examination of the specimens (Sayed et al., 2018).

###### Susceptibility test

*Candida albicans* ATCC 60193 was used as the test organism in the experiment. A parallel design of three groups, each having three randomly chosen rabbits (A total of nine rabbits, *n* = 3 per group) was employed. The experiment was performed as described before with slight modifications (Basha et al., 2013). Briefly, fifty microliters of each of the tested formulae (OE, OE_zero_ and VCZ suspension) were instilled in the lower conjunctival sac of the rabbit’s right eye using micropipette. In each rabbit, no drug was instilled in the left eye to serve as the control. At specific time intervals (1–10 h), four sterile filter paper disks (Whatman no.5, 6 mm in diameter) were wetted by placing the disks under the eyelid of each eye of each rabbit. For each eye (right and left), two disks were placed in a 1.5 ml Eppendorf tube containing 500 μl Sabouraud dextrose broth (SDB) inoculated with 10% v/v yeast suspension (10^7^ CFU/ml). The other two disks were placed in a 1.5 ml Eppendorf tube containing 500 μl uninoculated SDB; this was used as a blank during measuring the optical densities. All the tubes were then incubated at 25 ± 2 °C for 24 h under aerobic conditions. After incubation, 200 μl of each tube was transferred to a sterile 96-well plate and the optical densities (OD_600nm_) were read on an automated spectrophotometric plate reader (Biotek, Synergy 2, Winooski, VT, USA) at a single wavelength of 600 nm. The results were expressed as average growth inhibition % (mean ± SD).

The growth inhibition % was calculated using the following equation:

(3) $$\text{Growth inhibition \%}=\frac{\mathrm{Control}\;(\text{left eye})OD_{600\mathrm{nm}}-\text{ Test }(\text{right eye}){\text{ OD }}_{600\mathrm{nm}}}{\mathrm{Control}\;(\text{left eye})OD_{600\mathrm{nm}}}\;\times \;100$$

## Results and discussion

### Preparation of VCZ ultradeformable elastosomes by modified ethanol injection method according to 3^3^ Box-Behnken design

The preparation of VCZ ultradeformable elastosomes by ethanol injection method was successful. The resultant dispersions appeared yellowish in color due to their PC content. The 3^3^ Box-Behnken design resulted in 15 formulae, three of which were center points. The formulae were given the codes (E1 – E15). The composition and measured responses of the fifteen experimental runs are shown in Table 2. Because ZP and PDI values were acceptable across the whole design space, these two responses were removed from the optimization process.

**Table 2.** Experimental runs, independent variables and measured responses of voriconazole ultradeformable elastosomes.

Formula^a^	Composition			Y_1_: EE %	Y_2_: PS (nm)	Y_3_: ZP (mV)	Y_4_: PDI
	X_1_: PC: EA ratio	X_2_: Hyaluronic acid % w/v	X_3_: PVA %				
E1	4.00	0.75	3.00	62.2 ± 0.7	431.6 ± 4.4	−47.9 ± 4.8	0.316 ± 0.05
E2	1.00	0.25	3.00	53.7 ± 2.7	310.8 ± 0.5	−41.8 ± 1.1	0.293 ± 0.06
E3	1.00	0.50	1.00	49.6 ± 2.6	333.7 ± 9.5	−45.7 ± 4.1	0.329 ± 0.04
E4	2.50	0.75	1.00	47.9 ± 1.2	435.2 ± 13.9	−50.6 ± 1.3	0.367 ± 0.09
E5^b^	2.50	0.50	3.00	55.5 ± 1.8	363.6 ± 9.1	−47.6 ± 0.5	0.315 ± 0.02
E6	4.00	0.50	5.00	67.9 ± 0.8	371.5 ± 7.7	−49.4 ± 1.9	0.341 ± 0.08
E7	4.00	0.50	1.00	65.4 ± 1.2	418.7 ± 28.3	−48.6 ± 3.9	0.303 ± 0.05
E8^b^	2.50	0.50	3.00	57.3 ± 5.5	357.3 ± 20.6	−46.7 ± 5.3	0.298 ± 0.05
E9	1.00	0.50	5.00	47.2 ± 1.7	321.6 ± 6.9	−42.8 ± 1.3	0.313 ± 0.03
E10	1.00	0.75	3.00	39.7 ± 2.4	342.3 ± 6.9	−46.5 ± 0.2	0.425 ± 0.07
E11	2.50	0.75	5.00	60.6 ± 0.1	427.3 ± 14.6	−49.9 ± 2.8	0.384 ± 0.12
E12^b^	2.50	0.50	3.00	51.1 ± 9.9	352.3 ± 20.8	−43.9 ± 6.2	0.346 ± 0.08
E13	2.50	0.25	5.00	67.4 ± 1.0	345.3 ± 28.9	−39.5 ± 1.2	0.193 ± 0.13
E14	2.50	0.25	1.00	64.9 ± 2.1	347.5 ± 5.6	−42.5 ± 0.9	0.197 ± 0.06
E15	4.00	0.25	3.00	72.8 ± 0.3	375.5 ± 3.1	−40.3 ± 0.5	0.231 ± 0.03

Presented values are the mean ± SD (*n* = 3).

PC: phosphatidyl choline; EA: edge activator; PVA: polyvinyl alcohol; EE%: entrapment efficiency percentage; PS: particle size; ZP: zeta potential; PDI: polydispersity index.

^a^ All formulae contained 40 mg VCZ and a total of 500 mg of PC + EA, in a volume of 10 ml. The drug content of formulae ranged from 95.3 to 102.7 %.

^b^ Center points.

### Evaluation of the prepared ultradeformable elastosomes

#### Zeta potential (ZP)

Table 2 contains the ZP values for all formulations, which ranged from a minimum of −39.5 ± 1.2 to a maximum of −50.6 ± 1.3 mV (in terms of absolute value). All of the prepared ultradeformable elastosomes exhibited acceptable ZP values, which was an indication of the physical stability of dispersions (Shamma & Elsayed, 2013; Fahmy et al., 2019). Incorporation of HA led to highly negative ZP. This could be explained in the light of the fact that the incorporation of negatively charged HA causes extensive adsorption on the elastosomal surface. This result is supported by Wadhwa et al. (2010), who reported that incorporating HA decreased ZP of chitosan nanoparticles. Also, Tran et al. (2014) reported that increasing HA concentration first neutralized, then inversed the zeta potential of positively charged solid lipid nanoparticles.

#### Polydispersity index (PDI)

It is well known that PDI values close to 0 indicate size homogeneity, while values close to 1 signify sample heterogeneity (Zeisig et al., 1996). The recorded values (Table 2) ranged from 0.193 ± 0.13 to 0.425 ± 0.07. These concluded the relative homogeneity of the measured samples (Aburahma & Abdelbary, 2012).

#### Statistical analysis of the Box-Behnken design

Adequate precision assures the ability of model to navigate the design space when the measured signal to noise ratio is greater than 4, which was observed in both remaining responses (Y_1_: EE% and Y_2_: PS) (De Lima et al., 2011; Sayed et al., 2020). Also, the lack of fit was non-significant. On the other hand, the predicted R^2^ is a measure of design’s ability to predict values of different responses (Chauhan & Gupta, 2004). In both responses of our study, predicted and adjusted R^2^ values were in acceptable agreement (Table 3), ensuring there were no problems with the data or the model (Kaushik et al., 2006; Annadurai et al., 2008).

**Table 3.** Output data of the Box-Bhenken design analysis of voriconazole-loaded ultradeformable elastosomal formulae.

Response	Y_1_: Entrapment efficiency %	Y_2_: Particle size (nm)
Minimum	39.7	310.7
Maximum	72.8	435.2
Ratio	1.83	1.40
Model	Linear	Reduced Quadratic
R-squared	0.8839	0.8842
Adjusted R-squared	0.8523	0.8378
Predicted R-squared	0.7889	0.7237
Adequate Precision	17.02	14.404
PRESS value	259.24	6433.84
Significant factors	X_1_, X_2_	X_1_, X_2_
Regression equation (in terms of coded factors)^a^	EE = +57.56 +9.77 × A −6.05 × B +1.91 × C	PS = +351.53 +36.12 × A +32.17 × B +18.15 × B^2^14.49 × C^2^

^a^ A: Phosphatidyl choline: edge activator (PC: EA) ratio, B: hyaluronic acid % w/v, C: Polyvinyl alcohol % (from the PC: EA ratio).

#### Analysis of EE% model

**Figure 1.**
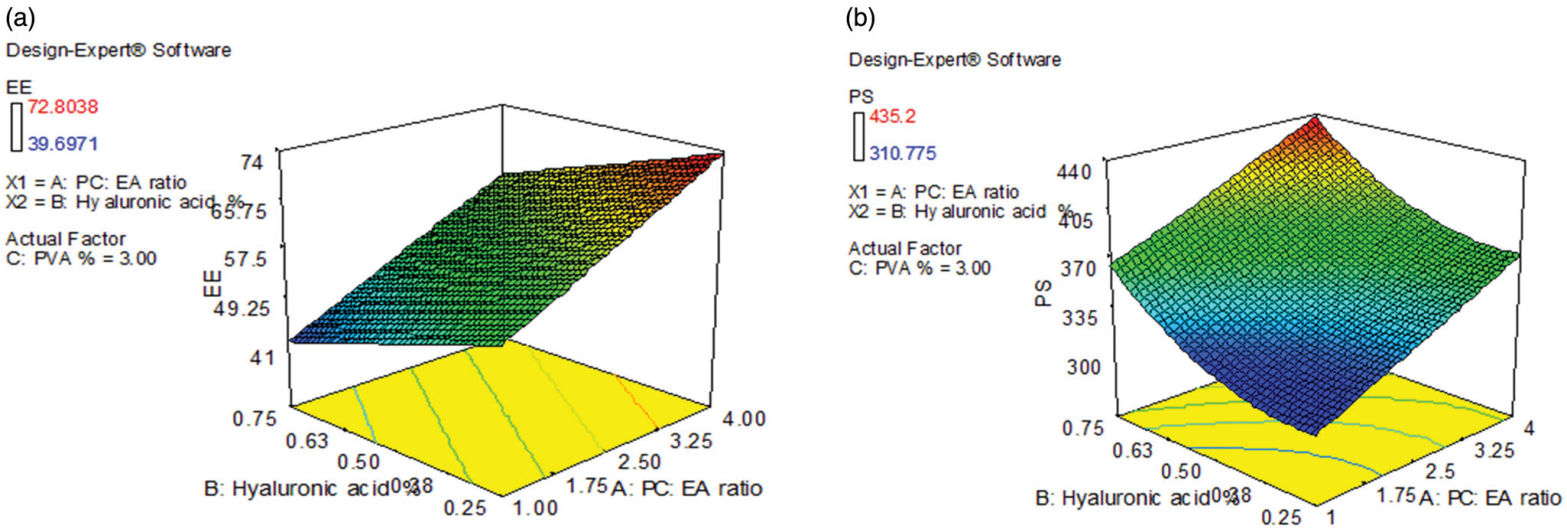
The effect of significant formulation variables on: (a) entrapment efficiency % and (b) particle size of voriconazole ultradeformable elastosomes.

Table 2 contains the EE% values for all formulations, which ranged from 39.7 ± 2.4 to 72.8 ± 0.3%. The output data (Table 3) and Figure 1(a) show the effect of the studied variables on the EE% of the ultradeformable elastosomal formulae. Both PC: EA ratio (X_1_) and HA % (X_2_) significantly affected the EE% of the prepared formulae (*p* < .0001 for PC: EA ratio and *p* = .0006 for HA %). On the other hand, PVA % (X_3_) had no significant effect (*p* = .1624).

Increasing the concentration of EA (brij S100) usually destabilizes the membrane of vesicles via pore formation, thus adversely affecting EE% (Fang et al., 2001; Gupta et al., 2005; Duangjit et al., 2011; Salama et al., 2012). In addition, elevated levels of EA may solubilize some of the drug causing it to diffuse into the aqueous phase in the preparation process. The solubilization of drug may occur when the EA concentration reaches its critical micelle concentration, where micelles begin to form and solubilize the drug preventing further entrapment within the vesicles, thus decreasing the total amount entrapped (van den Bergh et al., 2001; Basha et al., 2013). These suggestions were supported by Al-Mahallawi et al. (2014), who found that increasing sodium cholate concentration negatively affected the EE% of ciprofloxacin in transfersomes.

Increasing the concentration of HA may lead to interaction with and partial disruption of the vesicle membrane bilayer leading to the diffusion of drug outside the vesicles. This finding agrees with Wadhwa et al. (2010), who reported that increasing sodium hyaluronate ratio decreased the EE% of dorzolamide hydrochloride and timolol maleate in chitosan nanoparticles.

#### Analysis of PS model

Table 2 contains the PS values for all formulations, which ranged from 310.8 ± 0.5 to 435.2 ± 13.9 nm. The output data (Table 3) and Figure 1(b) show the effect of the studied variables on the PS of the ultradeformable elastosomal formulae. Both PC: EA ratio (X_1_) and HA % (X_2_) significantly affected the PS of the prepared formulae (*p* = .0002 for PC: EA ratio and *p* = .0006 for HA %). On the other hand, PVA % (X_3_) had no significant effect (*p* = .2229).

The direct relation between PC: EA ratio and PS can be due to the lowering of interfacial tension at elevated levels of EA (brij S100), accompanied by an increase of its ability to emulsify the ingredients used. Additionally, elevated EA levels can favor the formation of micelles in the medium, thus lowering the average PS (van den Bergh et al., 2001; Salama et al., 2012; Basha et al., 2013). Moreover, using lower proportions of EA may not be enough for the full coverage of the vesicular surface, thus increasing the tendency of vesicles agglomerate to decrease their total surface area making the EA sufficient to fully coat the surface of the formed agglomerate. This finally yields a relatively stable dispersion of higher PS (Dora et al., 2010; Ghorab et al., 2011). On the other hand, the direct relation between the vesicular size and PC concentrations can be due the hindrance of interaction between the polar heads of the EA molecules (Junyaprasert et al., 2012). These findings were similar to Al-Mahallawi et al. (2014) and Salama et al. (2012) upon the preparation of ciprofloxacin and olanzapine transfersomes, respectively.

The direct relation between HA % and PS can be due to the adsorption of HA (negatively charged) by electrostatic attraction to the positively charged nitrogen atom of PC in the vesicular wall of ultradeformable elastosomes forming a coating layer that increased the PS with increase in HA concentration. This agrees with Tran et al. (2014), who reported that increasing hyaluronic acid concentration dramatically increased the PS of vorinostat solid lipid nanoparticles. Wadhwa et al. (2010) also reported a direct relation between HA concentration and the PS of chitosan nanoparticles.

#### Optimization of VCZ ultradeformable elastosomes based on the desirability criterion

The optimum values of the variables were obtained by numerical optimization based on the criterion of desirability using the Design-Expert-7^®^ software, taking into account that PVA concentration was maximized to get benefit of its stabilizing property (Basalious et al., 2010). A suggested optimal formula composed of PC and brij S100 at the weight ratio of 3.62: 1, 0.25%w/v HA and 5% (percentage from the PC/brij S100 mixture) PVA had the highest desirability value (0.916). This formula was prepared, and its observed responses were compared to the predicted ones. The high similarity between the observed and predicted responses of OE (Table 4) could lead to the conclusion that the elucidated models were valid.

**Table 4.** The predicted and the observed responses of the optimal voriconazole ultradeformable elastosomal formula (OE).

Response^a^	Entrapment Efficiency %	Particle size (nm)	Zeta potential (mV)	Polydispersity index
OE Observed value	72.6	362.4	−41.7	0.250
OE Predicted value	72.8	355.0	–	–

^a^ Optimal ultradeformable elastosomal formula was composed of phosphatidyl choline and brij S100 at the weight ratio of 3.62: 1, 0.25 %w/v hyaluronic acid and 5% (percentage from the phosphatidyl choline/ brij S100 mixture) polyvinyl alcohol.

#### Further *in vitro* characterization of optimal ultradeformable elastosomal formula

##### Measurement of the deformability index (DI) of optimal ultradeformable elastosomes

DI values of OE and the corresponding liposomes formula were 12.8 ± 0.4 and 8.7 ± 0.4 g, respectively. OE has shown to be significantly more elastic than the corresponding liposomal formula *(p* < .05).

The inclusion of hydrophilic EAs (brij S100 in our study) boosts the elasticity of vesicles via destabilization of their membranes and creation of systems with disrupted packing characteristics through significant lowering of interfacial tension, allowing these ultradeformable vesicles of larger size to squeeze through them more easily than conventional liposomes (Trotta et al., 2002; Kakkar & Kaur, 2011).

##### Effect of short-term storage

The values of drug content, EE%, PS, ZP, PDI and DI for fresh and stored OE (3 months) are listed in Table 5. No statistical difference was found in drug content, EE%, PS, ZP, PDI and DI (*p* > .05 for all values). Moreover, there was no change in the physical appearance of the formula. All these suggest that OE exhibited acceptable stability.

**Table 5.** Effect of storage on different measurements of OE and its PVA lacking analogue.

Parameter*	Fresh OE	Stored OE (3 months)	Fresh OE lacking PVA	Stored OE lacking PVA (3 months)
Drug content	98.8 ± 0.9^a^	96.7 ± 0.7^a^	–	–
Entrapment efficiency %	72.6 ± 0.2^a^	72.2 ± 0.3^a^	–	–
Particle size (nm)	362.4 ± 10.0^a^	365.5 ± 12.3^a^	299.6 ± 1.3**^a^**	293.6 ± 3.0**^a^**
Zeta potential (mV)	−41.7 ± 2.7^a^	−39.9 ± 0.1^a^	−37.5 ± 1.0**^a^**	−22.6 ± 1.3**^b^**
Polydispersity Index	0.25 ± 0.06^a^	0.28 ± 0.08^a^	0.28 ± 0.00**^a^**	0.29 ± 0.03**^a^**
Deformability index (g)	12.8 ± 0.4^a^	14.9 ± 0.3^a^	–	–

Different superscripts indicate significant difference. Normal superscripts are used for the stored OE formula and its fresh counterpart, while bolded superscripts are reserved for the stored PVA lacking analogue and its fresh counterpart..

*Mean ± SD (*n* = 3).

OE: Optimal ultradeformable elastosomal formula; PVA: polyvinyl alcohol.

Table 5 shows also the values of PS, ZP and PDI for the OE lacking PVA, before and after storage for 3 months. No significant difference was detected except for the value of ZP (at *α* = 0.05).

Although the measured PS was not significantly different from the fresh formula, the decrease in absolute value of ZP of OE lacking PVA was accompanied by visible aggregation of the particles and decrease in the sediment volume after storage, with very difficult redispersion. The formula had to be shaken for several times before it restored its homogeneity. This is a sign of physical instability that could lead to non-uniform dosing with every use. The role of PVA is to prevent such events by coating the surface of particles, maintaining the negative zeta potential and dispersion of particles, and thus physical stability of the formula (Al-mahallawi et al., 2019). This is evident by the significantly smaller PS of vesicles lacking PVA (*p* < .05).

##### Morphology of VCZ optimal ultradeformable elastosomes

**Figure 2.**
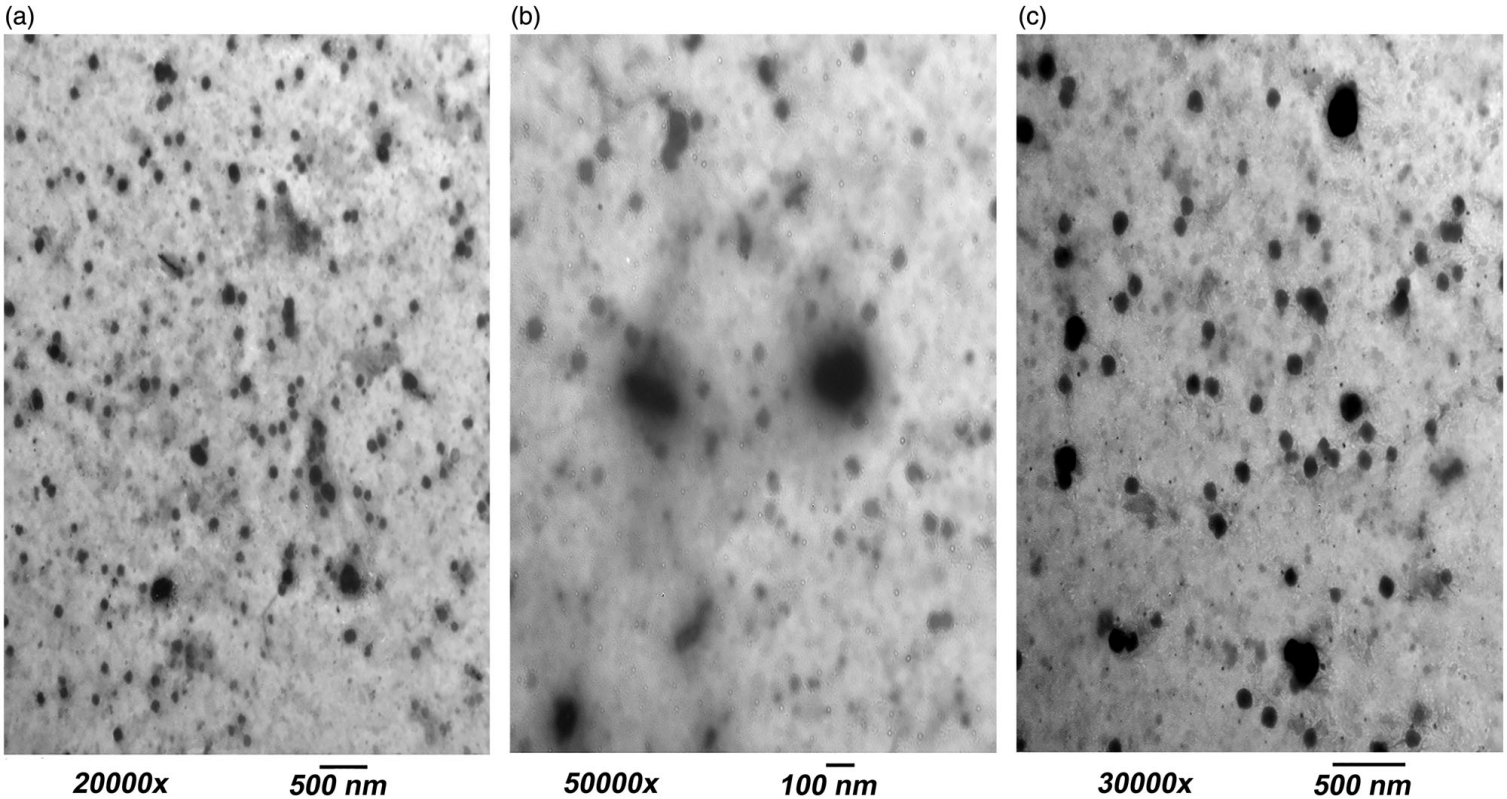
Transmission electron microscope (TEM) micrographs showing: (a) OE vesicles, (b) a close-up on OE vesicles demonstrating an outer transparent shell of hyaluronic acid and (c) OE_zero_ vesicles.

The TEM micrographs of OE are shown in Figure 2(a,b). It is demonstrated that the nanovesicles are present in a nearly perfect spherical shape. However; the PS was smaller than determined by the dynamic light scattering. The difference may be attributed to the method of measurement. Dynamic light scattering measures the average PS (Z-average), while TEM measures the individual PS (Dahiya et al., 2018). Figure 2(c) demonstrated the spherical morphology of OE_zero_ vesicles. It is worth mentioning that OE had and extra transparent outer layer ‘shell’ confirming that HA coats the outer surface of vesicles and accounting for the increase in PS by increasing the concentration of HA (Figure 2(b)) (Tran et al., 2014).

##### pH measurement

pH values of OE and OE_zero_ were found to be 7.37 ± 0.02 and 7.39 ± 0.01, respectively, implying the suitability of their ocular use (pH of tears =7.4) (Achouri et al., 2013).

##### Refractive index (RI)

RI values of OE and OE_zero_ were found to be 1.3380 and 1.3363, respectively, which is within the acceptable range (less than 1.5), causing no discomfort or blurred vision (Ammar et al., 2009).

##### *In vivo* evaluation of OE and OE_zero_ in New Zealand white rabbits

###### Safety (Histopathological testing)

**Figure 3.**
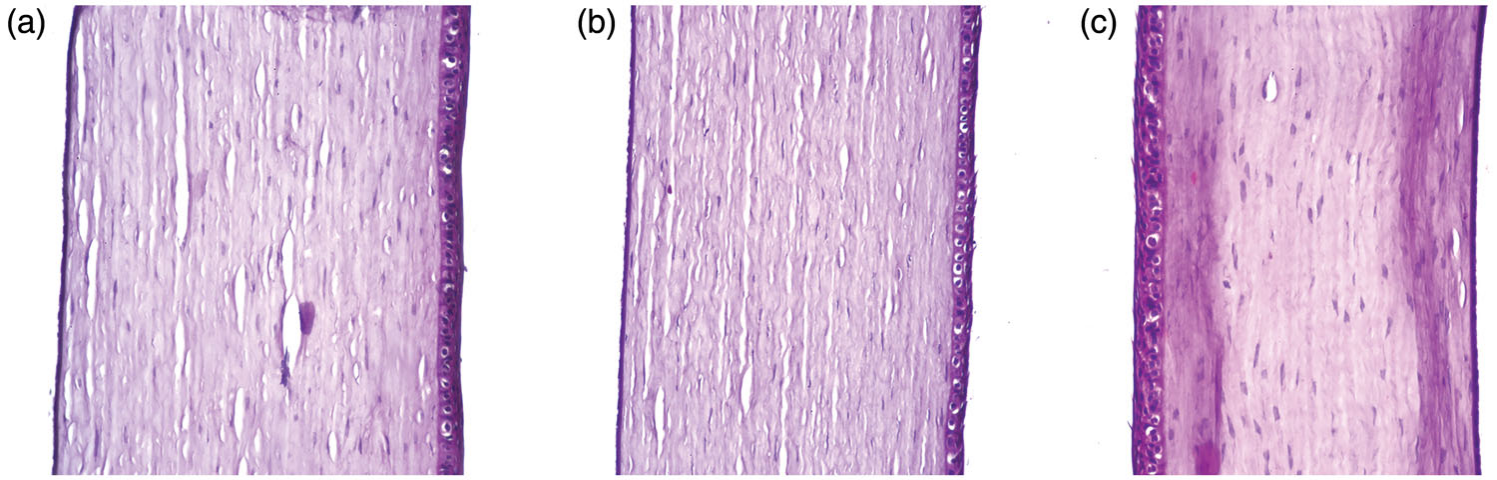
Microscopic photographs showing normal histological structure of rabbit corneas treated with (a) normal saline (negative control), (b) OE and (c) OE_zero_.

It is very important to test for the biocompatibility of the used components for ocular application. Unlike the other ingredients used in our study, and to our best of knowledge, this was the first time brij S100 was used in ocular formulations. The Examination of photomicrographs (Figure 3(a–c)) revealed that the exposure of rabbit corneas to normal saline as a negative control (Figure 3(a)), OE (Figure 3(b)) or OE_zero_ (Figure 3(c)) did not cause any sign of irritation. All corneas conserved normal histological structure including all the corneal layers: the epithelium, the stroma and the endothelium. Then it can be concluded that OE and OE_zero_ can be safely applied to the eye.

###### Susceptibility test

**Figure 4.**
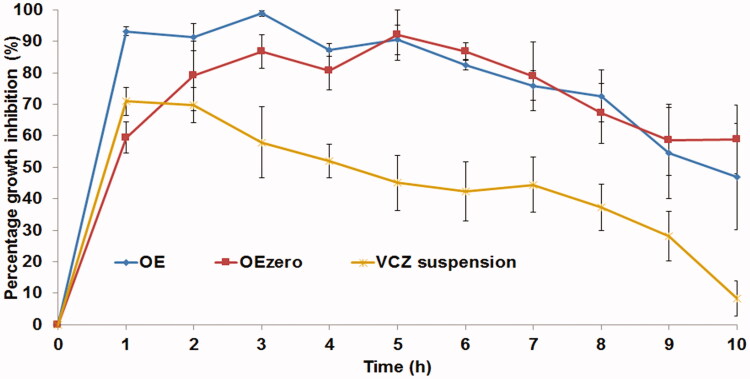
Percentage inhibition of *C. albicans* growth produced by voriconazole-loaded formulae compared to voriconazole suspension in rabbit external ocular tissue.

The antifungal activity of OE and OE_zero_ was compared to that of VCZ suspension. The percentage growth inhibition of *C. albicans* produced by different formulae was plotted against time (Figure 4) .The area under the inhibition time curve (AUC_0–10h_) (% growth inhibition.h) was calculated from the curve of each individual animal using Kinetica® 2000 software using non-compartmental extravascular analysis, applying trapezoid rule. One-way ANOVA, followed by Duncan’s post hoc test were performed to compare the average AUC_0–10h_ values resulting from VCZ suspension, OE and OE_zero_. Figure 4 illustrates that the antifungal effect of VCZ suspension showed a maximum at 1 h post administration and decreased gradually afterwards. In contrast, OE and OE_zero_, showed almost a constant antifungal effect for about 8 h, reaching their maxima at 3 and 5 h, respectively, signifying better corneal retention. The AUC_0–10h_ values of OE and OE_zero_ were 768.8 ± 61.3 and 683.1 ± 115.1% growth inhibition.h, respectively, which was significantly higher (*p* = .008) than that of VCZ suspension (432.1 ± 75.8% growth inhibition.h), with folds increase in AUC_0–10h_ equal to 1.78 and 1.58 for OE and OE_zero_, respectively. This probably was caused by the smaller PS of the test formulae when compared to VCZ suspension, leading to longer residence times and more effective corneal contact of VCZ, agreeing with what was previously noted by Kassem et al. (2007) during the fabrication of glucocorticoid nanosuspensions. Moreover, the formulation components (Brij S100, HA and PVA) are known to have excellent interaction with the corneal surface, favoring prolonged corneal contact time and thus leading to resistance of the formulae to the effect of blinking and tear film turn over (Hao et al., 2002; Sayed et al., 2020). Regarding the comparison between the investigated formulae themselves (OE and OE_zero_), Duncan’s post hoc test showed there were no significant results upon comparing the AUC_0–10h_ values. However, there is a numerical superiority of OE to its HA-lacking analogue OE_zero_, possibly due to the mucoadhesive properties of HA. Hence, VCZ-loaded ultradeformable elastosomes exhibit a higher ability for precorneal retention when compared to drug suspension (Basha et al., 2013), favoring them for ocular use, with sustained drainage from the conjunctival sac and almost no ocular discomfort.

## Conclusion

The fabrication of VCZ ultradeformable elastosomes using modified ethanol injection method, according to a 3^3^ Box-Behnken design was successful. A suggested optimal formula composed of PC and brij S100 at a weight ratio of 3.62: 1, 0.25%w/v HA and 5% (percentage from the PC/brij S100 mixture) PVA had the highest desirability value. There was an agreement between the predicted and observed responses. The optimized formula had significantly higher elasticity than conventional liposomes. It maintained acceptable stability upon refrigeration at 4–8 °C for three months. The vesicles demonstrated a nearly perfect spherical morphology with an external coat of HA upon visualization using TEM. The optimized formula was expected to cause no ocular irritation or blurring in vision by having suitable values of pH and RI. The histopathological study revealed safety of the optimized formula for ocular use due to the protective effect of hyaluronic acid. The fungal susceptibility to VCZ using *C. albicans* proved the superiority of the optimized formula to VCZ suspension, with higher and more durable growth inhibition. Therefore, it can be concluded that the optimized VCZ-loaded ultradeformable elastosomes can be a promising treatment for fungal keratitis.
